# Optimal deep brain stimulation site and target connectivity for chronic cluster headache

**DOI:** 10.1212/WNL.0000000000004646

**Published:** 2017-11-14

**Authors:** Harith Akram, Sarah Miller, Susie Lagrata, Marwan Hariz, John Ashburner, Tim Behrens, Manjit Matharu, Ludvic Zrinzo

**Affiliations:** From the Unit of Functional Neurosurgery (H.A., M.H., L.Z.), Sobell Department of Motor Neuroscience and Movement Disorders, and Wellcome Trust Centre for Neuroimaging (J.A., T.B.), UCL Institute of Neurology, University College London; Victor Horsley Department of Neurosurgery (H.A., L.Z.), National Hospital for Neurology and Neurosurgery; Headache Group (S.M., S.L., M.M.), UCL Institute of Neurology and National Hospital for Neurology and Neurosurgery, London, UK; Department of Clinical Neuroscience (M.H.), Umeå University, Sweden; and Centre for Functional MRI of the Brain (T.B.), John Radcliffe Hospital, Oxford, UK.

## Abstract

**Objective:**

To investigate the mechanism of action of deep brain stimulation for refractory chronic cluster headache and the optimal target within the ventral tegmental area.

**Methods:**

Seven patients with refractory chronic cluster headache underwent high spatial and angular resolution diffusion MRI preoperatively. MRI-guided and MRI-verified electrode implantation was performed unilaterally in 5 patients and bilaterally in 2. Volumes of tissue activation were generated around active lead contacts with a finite-element model. Twelve months after surgery, voxel-based morphometry was used to identify voxels associated with higher reduction in headache load. Probabilistic tractography was used to identify the brain connectivity of the activation volumes in responders, defined as patients with a reduction of ≥30% in headache load.

**Results:**

There was no surgical morbidity. Average follow-up was 34 ± 14 months. Patients showed reductions of 76 ± 33% in headache load, 46 ± 41% in attack severity, 58 ± 41% in headache frequency, and 51 ± 46% in attack duration at the last follow-up. Six patients responded to treatment. Greatest reduction in headache load was associated with activation in an area cantered at 6 mm lateral, 2 mm posterior, and 1 mm inferior to the midcommissural point of the third ventricle. Average responders' activation volume lay on the trigeminohypothalamic tract, connecting the trigeminal system and other brainstem nuclei associated with nociception and pain modulation with the hypothalamus, and the prefrontal and mesial temporal areas.

**Conclusions:**

We identify the optimal stimulation site and structural connectivity of the deep brain stimulation target for cluster headache, explicating possible mechanisms of action and disease pathophysiology.

Long-term, high-frequency deep brain stimulation (DBS) in the ventral tegmental area (VTA) has been shown to be a safe and effective treatment modality for patients with refractory chronic cluster headache (CCH).^1–3^

The underlying pathophysiology in cluster headache (CH) is not fully understood.^4^ The hypothalamus has been implicated in the disease process,^5^ and pathologic activation of the trigemino-parasympathetic brainstem reflex is thought to be responsible for simultaneous activation of trigeminal nerve and craniofacial parasympathetic nerve fibers, respectively, leading to the characteristic ipsilateral cranial pain and autonomic features.^6,7^

The periodicity of individual attacks, the relapsing-remitting course, and the seasonal recurrence of headache bouts are suggestive of hypothalamic involvement.^7^ This is supported by neuroendocrinological^8^ and neuroimaging studies.^5,9^ The area referred to in the neuroimaging studies has been described as the posterior hypothalamus, although the locus of maximum activation lies in the VTA.^3,10^

The exact mode of action of DBS for CH and the neural networks involved remain poorly understood. Furthermore, the optimal stimulation site is yet to be identified.^2,10–12^ Activation of the trigeminal nerve and ganglion has been demonstrated with hypothalamic stimulation,^13^ possibly mediated by the trigeminohypothalamic tract (THT) described in nonhuman studies.^14,15^

The objectives of this study were to identify the optimal VTA stimulation site for improvement in headache load (HAL) and to explore the connectivity or fingerprint of stimulation tissue volumes in responders to identify the THT by proceeding through the following steps: generating volume of tissue-activated models for all active DBS contacts, carrying out a voxel based morphometry–style regression analysis of modeled activation volumes and their associated efficacy profiles, and performing tractography from modeled activation volumes of active DBS contacts in responders using a probabilistic approach and state-of-the-art high angular resolution diffusion imaging.

## METHODS

### Standard protocol approvals, registrations, and patient consents.

This study received ethics approval from the West London NHS Research Ethics Committee (10/H0706/68). All patients provided written consent.

### Patients.

Seven patients (5 male) were recruited. Five patients belonged to a cohort that has been published previously concerning the efficacy and safety of DBS for CCH.^3^ All patients fulfilled the International Classification of Headache Disorders-2 and -3beta diagnostic criteria for CCH and had experienced highly disabling, medically refractory symptoms for at least 2 years.^16,17^ All patients had failed to respond to medical therapy trials. Selection criteria have been described elsewhere.^3^ Inclusion in the present study was limited to patients who could tolerate lying flat for the duration of the preoperative scan and who had no contraindications to 3T MRI.

### Preoperative MRI data acquisition.

Imaging pertinent to this study was performed before surgery on a 3T Siemens Magnetom-Trio (Erlangen, Germany) with a 32-channel receive head coil. Padding was used inside the head coil to reduce discomfort and head motion.

### Diffusion-weighted MRI.

Diffusion images were acquired with a Siemens 511E Advanced Echo Planar Imaging Diffusion WIP. In-plane acceleration was used (generalized autocalibrating partially parallel acquisitions factor of 2) with partial Fourier 6/8. In-plane resolution was 1.5 × 1.5 mm^2^ (field of view 219 × 219 mm^2^, repetition time 12,200 milliseconds, echo time 99.6 milliseconds), and 85 slices were acquired with a 1.5-mm thickness. Diffusion weighting with b = 1,500 s/mm^2^ was applied along 128 directions uniformly distributed on the sphere, and 7 b = 0 seconds volumes were also acquired. To correct for distortions, all acquisitions were repeated with a reversed phase-encoding direction (left-to-right and right-to-left phase encode), giving a total of 270 volumes acquired ([128 + 7] × 2). Total acquisition time was 62 minutes.

The surgical procedure, intraoperative MRI acquisition, and postoperative DBS programming have been previously described elsewhere.^3^ This included stereotactic magnetization-prepared rapid gradient-echo (MPRAGE) images before and after DBS lead implantation.

### Outcome measures and follow-up.

Outcome data were collected and recorded prospectively. These included daily attack frequency, attack severity, attack duration, HAL, and adverse events (including surgical complications, stimulation-induced adverse events, and morbidity). Headache severity was measured on the verbal rating scale for pain (0 being no pain and 10 being the worst pain imaginable). Attack frequency was defined as the number of CH attacks per day and duration as the time in hours of each recorded attack. The individual scores from both were then averaged over the 2-week observation period. HAL was defined as Σ (severity [on the verbal rating scale]) × (duration [in hours]) of all headache attacks occurring over a 2-week period. We introduced this measure previously and suggested that it effectively reflects response to treatment.^3^ These measures were assessed with 2-week headache diaries collected preoperatively (baseline); at the beginning of DBS therapy; at 3, 6, and 12 months; and yearly thereafter.

Responders were defined as patients with sustained HAL reduction ≥30% because this was deemed meaningful in line with the Initiative on Methods, Measurement, and Pain Assessment in Clinical Trials guidelines.^18^

### Analysis of activation volumes.

#### DBS contacts volume of tissue-activated modeling.

SureTune (Medtronic Inc, Minneapolis, MN), a DBS therapy planning platform, was used to model activation volumes around individual contacts. The platform applies neuron models coupled to finite-element simulations as described by Åström and colleagues^19^ to generate DBS therapy activation volumes. After implantation, stereotactic MPRAGE scans were coregistered with preimplantation stereotactic MPRAGE scans and realigned with a plane parallel to the anterior commissure–posterior commissure (AC-PC) line.

The postimplantation MPRAGE was used to fit the DBS lead model within the MRI artifact produced by the leads. Activation volumes were generated around active DBS contacts with corresponding voltages.

#### Interparticipant alignment.

Preimplantation MPRAGE scans were brain extracted with the Brain Extraction Tool (FSL version 5.0). A 2-step procedure was used to register native scans to the Montreal Neurological Institute (MNI) 152 standard-space T1-weighted average structural template image (1-mm resolution). The first step used a linear (affine) transformation with FLIRT (Centre for Functional MRI of the Brain's Linear Image Registration Tool) using 12 *df*. The output from this step was used to execute nonlinear registration (second step) with FNIRT (Centre for Functional MRI of the Brain's Non-Linear Image Registration Tool). This process produced individual native-to-standard (MNI space) nonlinear warp fields that were then applied to the DBS activation volumes acquired from SureTune to transform all volumes to standard space.

#### Average DBS activation volume and efficacy cluster.

All lateralized volumes (right-sided volumes were flipped to left) were merged with Fslmerge (FSL version 5.0) into a 4-dimensional data file. A single-group average (1-sample *t* test) general linear model design was used to test against percentage improvement in HAL.

Nonparametric permutation inference was then carried out on each voxel with Randomise (FSL version 5.0) with 5,000 permutations to build up the null distribution to test against. Percentage improvement in HAL was demeaned, and single-group *t* test with threshold-free cluster enhancement was used as the test statistic. Cluster-based inference with Cluster (FSL version 5.0) was carried out to extract the clusters and local maxima in outputs.

### Tractography from modeled activation volumes.

See the supplemental material at Neurology.org for diffusion preprocessing.

Probabilistic tractography was generated in ProbtrackX2 GPU version (FSL version 5.0) (number of samples 5,000, curvature threshold 0.2, step length 0.5 mm, subsidiary fiber volume fraction threshold 0.01). The process repetitively samples from the distributions of voxel-wise principal diffusion directions generated in BedpostX, each time computing a streamline through these local samples to generate a “probabilistic streamline” or a sample from the distribution on the location of the true streamline, building up a spatial “connectivity distribution.” Streamlines truly represent paths of minimal hindrance to diffusion of water in the brain, but they are reasonable indirect estimates of long-range white matter connections.^20^

Probabilistic tractography was generated for all responders with the DBS activation volume as seeds and the cerebellum and contralateral hemisphere as the exclusion mask. CSF termination masks were used to exclude false-positive streamlines.

## RESULTS

### Patients.

Scanning and surgery proceeded with no adverse effects. The mean (SD) follow-up was 33 (14) months (median 34 months). Six patients responded to DBS. The patient who did not respond (CH2) was also the only patient to have received occipital nerve stimulation before DBS. This was removed after 5 years for lack of response. There was no surgical morbidity or mortality. Two patients with side-alternating attacks underwent bilateral surgery in 1 episode, bringing the total to 9 implanted DBS leads in this series. Table 1 shows demographics, disease duration, length of follow-up, stimulation amplitudes, and change in HAL, attack severity, attack frequency, and attack duration at the final follow-up from baseline, along with patient's estimated percentage of improvement after surgery.

**Table 1:**
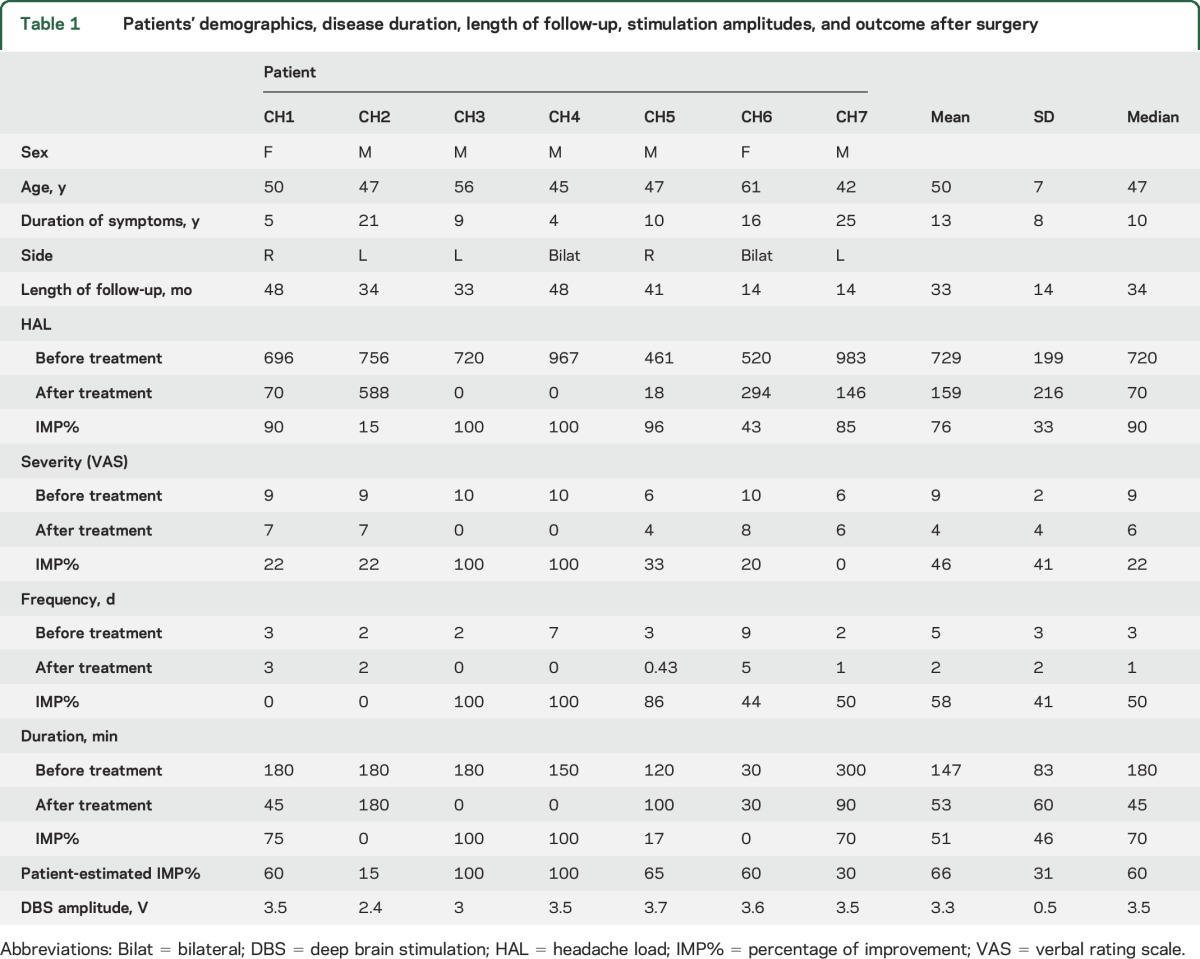
Patients' demographics, disease duration, length of follow-up, stimulation amplitudes, and outcome after surgery

### Stimulation-induced adverse events.

There were no serious adverse effects from DBS. Two patients developed transient dizziness; 1 patient developed nausea; and 1 patient developed intermittent diplopia. All were improved with adjustment of the stimulation amplitude. One patient (CH2) developed troublesome diplopia, oscillopsia, and nystagmus with DBS amplitudes >2 V.

### DBS activation volume modeling and efficacy cluster.

**Figure 1:**
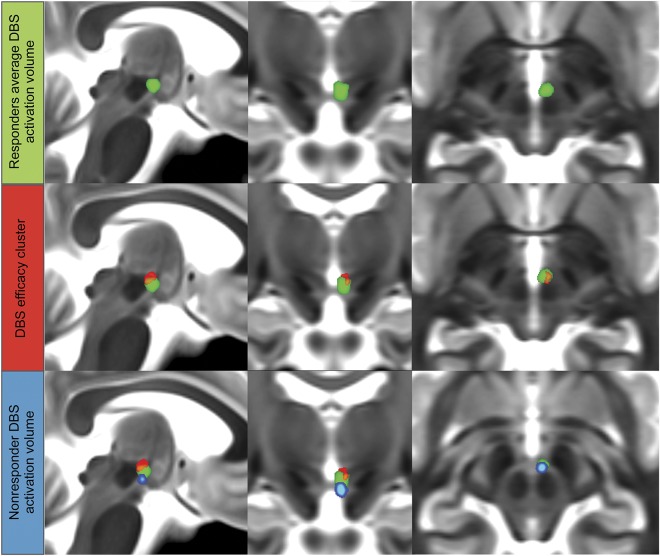
DBS activation volume and efficacy cluster Average DBS activation volume (green) with DBS efficacy cluster (red) and activation volume for the nonresponder (blue). DBS = deep brain stimulation.

Responders’ average DBS activation volume, nonresponder’s (CH2’s) DBS activation volume, and statistically significant cluster correlated to higher stimulation efficacy (improvement in HAL) are shown in figure 1. The responders' average activation volume lies in the VTA in the area between the red nucleus and the mammillothalamic tract. The cluster predictive of improvement in HAL lies in the superior, posterior, and lateral portion of the group average activation volume. The activation volume for the nonresponder lies outside the efficacy cluster. Table 2 give average activation and cluster volumes with MNI coordinates.

**Table 2:**
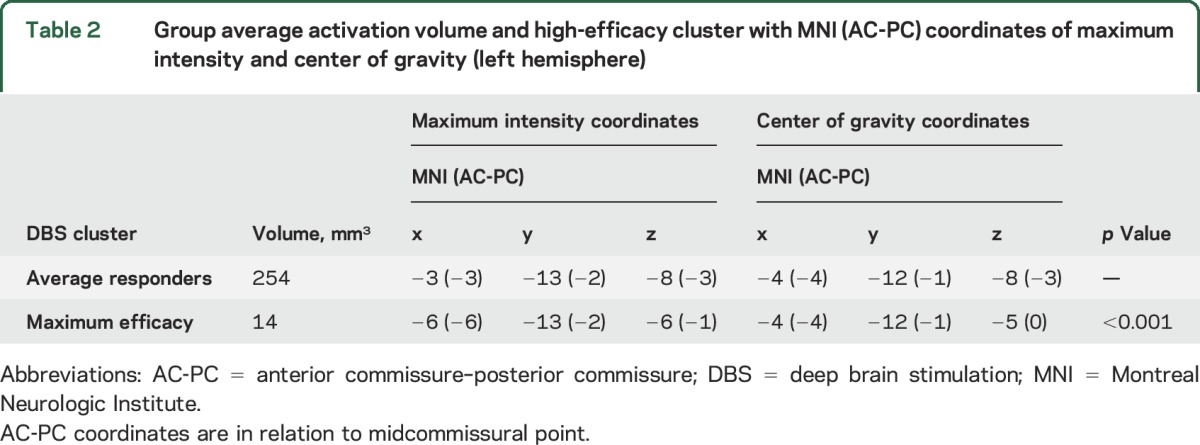
Group average activation volume and high-efficacy cluster with MNI (AC-PC) coordinates of maximum intensity and center of gravity (left hemisphere)

### Tractography.

**Figure 2:**
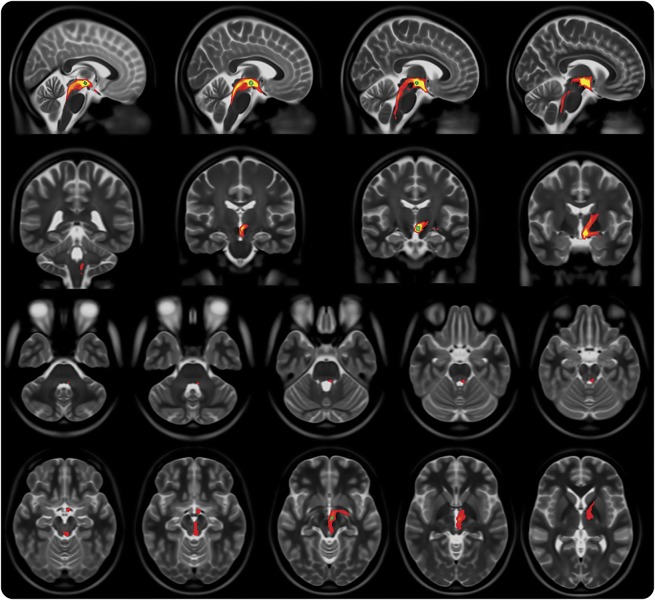
Probabilistic tractography streamlines Group average probabilistic tractography streamlines (red-yellow) with group average deep brain stimulation tissue activation volume (green).

Group average streamlines generated from individual responders’ DBS activation volume are shown in figure 2. Anteriorly, the streamlines traverse the hypothalamus and then split into 2 pathways: an inferolateral pathway toward the mesial temporal lobe and amygdalar complex, possibly via the amygdalofugal pathway, and an anterosuperior pathway toward the prefrontal area via the anterior limb of the internal capsule. Posteriorly, the streamlines run medial to the red nucleus toward the periaqueductal gray and then caudally through the pons and upper medulla in a dorsolateral position toward the trigeminal tract and nuclei.

## DISCUSSION

Voxel-based statistical analysis of active DBS contacts activation volumes at the last follow-up point after VTA DBS was used in 7 patients with medically refractory CCH to identify a statistically significant cluster in the stimulation area, reflecting the highest-efficacy zone. The responders' activation volumes (6 patients, 8 DBS contacts) were also used to generate probabilistic tractography streamlines to identify the THT.

We show that patients were appropriately selected (table 1) as demonstrated in disease duration and headache characteristics. Furthermore, 6 of 7 patients had indeed responded well to DBS as demonstrated by the improvement in HAL, duration, frequency, and severity.

The first patient^21^ and patient series^1^ to undergo DBS for CCH had the target in what was called the hypothalamic gray. The target came to light after a PET study found increased activation in this area in patients with CH during attacks.^9^ The target in this area, which we identify as the VTA, is not readily demarcated. This is due to 3 factors; first, the target has to be identified with the use of surrounding landmarks on MRI (e.g., red nucleus, mammillothalamic tract); second, the stimulation amplitude (an average of 3.3 V in this study) covers a comparatively large brain tissue area around the active DBS contact, hence allowing some leeway in targeting accuracy; and third, PET studies are subject to misalignment during the coregistration process, potentially introducing a spatial error.^22^ This has been reflected in the discrepancy in the reported coordinates of activation with another PET study^23^ and with an fMRI study.^24^

The coordinates of the original target were 2 mm lateral to the midline, 6 mm behind the midcommissural point (MCP) and 8 mm below the AC-PC.^21^ This is the same area identified in an earlier PET study.^9^ The target was then modified to 2 mm lateral to the midline, 3 mm posterior, and 5 mm below the MCP.^1^ This last Franzini target has generally been adopted by other groups.^12^

A study of 10 patients with CCH implanted with unilateral DBS leads using the Franzini target used postoperative AC-PC coordinates of the active DBS contact centers projected on the Schaltenbrand atlas and a 3-dimensional 4.7T MRI atlas of the diencephalon-mesencephalic junction atlas to identify the anatomic location of the effective DBS electrodes.^12^ Five patients responded to treatment. The mean coordinates of the active contacts in the responders were 3 mm lateral, 3.5 mm posterior, and 3.3 mm below the MCP. The study, however, did not find a statistically significant difference between the responder and nonresponder groups. The authors pointed out the limitation of the method used to localize the contacts, i.e., projection of AC-PC coordinates on atlases.^12^ These coordinates are within 1.5 mm from the coordinates of the average volume of DBS activation (maximum intensity point) in the responders in our study.

Our voxel-based morphometry regression analysis shows the coordinates of the higher-efficacy predictive cluster (maximum intensity point) to be further lateral and superior (6 mm lateral, 2 mm posterior, and 1 mm inferior to the MCP). This seems to be supported by the relation of the DBS activation volume of the single nonresponder in our study to the efficacy cluster lying outside it, as shown in figure 1.

The difficulty in explaining the mechanism of action of DBS in CH is partly caused by the lack of a definitive understanding of the pathophysiologic process itself.^25,26^ Some authors suggest that simple local blockade of the posterior hypothalamic gray or VTA activity is not a likely mechanism for improvement in headache. However, many patients experience a microlesion or stun effect with complete abolition of attacks for a few days or even weeks after DBS lead implantation alone, suggesting disruption of pathologic neural activity in the region.^3,7,25^ However, this does not explain the latency in achieving maximal DBS efficacy that has been seen across several studies, including our own. Increased threshold for cold pain at the site of the first trigeminal branch ipsilateral to the stimulated side in patients stimulated long-term could be caused by modulation of the antinociceptive system^27^; however, a generic antinociceptive effect does not explain why DBS is effective for the trigeminal autonomic cephalalgias but not atypical facial pain.^7,25,26^ DBS has been shown to modulate a complex network of pain-processing areas.^13^ Stimulation induced local activation around the active DBS contact and distant activation in the ipsilateral thalamus, somatosensory cortex and precuneus, anterior cingulate cortex, and ipsilateral trigeminal nucleus and ganglion, coupled with deactivation in the middle temporal gyrus, posterior cingulate cortex, inferior temporal gyrus bilaterally, and contralateral anterior insula.^13^ This study was the first to document a functional connection between the hypothalamus and the trigeminal system in humans in vivo. The activation in the trigeminal system, however, does not seem to provoke CH pain attacks or the typical sensations that commonly accompany trigeminal activation.^13^ This connection has been previously observed after injection of the neuropeptide orexin B into the posterior hypothalamic region of the rat, which increased spontaneous activity in the caudal trigeminal nucleus (with discharges persisting for several minutes) and heightened responses in the nucleus to dural stimulation and noxious thermal stimulation of the face.^15^

The connection between the trigeminal system and the hypothalamus is crucial in integrating somatosensory and visceral information (e.g., innervation from cranial skin, intracranial blood vessels, and meninges) with endocrine and autonomic responses.^14^ Single-unit recording and antidromic microstimulation techniques in rats have established a direct 2-way connection between the posterior hypothalamus and the spinal trigeminal nucleus through the THT.^14^

Other brainstem nuclei have neurons that respond to noxious and innocuous somatosensory and visceral stimulation. These nuclei also give efferents to the hypothalamus such as the parabrachial nuclei,^28^ nucleus of the solitary tract,^29^ periaqueductal gray,^30^ and caudal ventrolateral medulla,^31^ suggesting that somatosensory signals reach the hypothalamus through several polysynaptic pathways.^14^

Previous work has explored the structural connectivity of the DBS target using probabilistic tractography in healthy controls.^32^ Comparable connections to the frontal and temporal areas were described alongside connections to the periaqueductal gray. At the time, image acquisition parameters were not sufficient for accurate tracking in the brainstem, which we present here.

**Figure 3:**
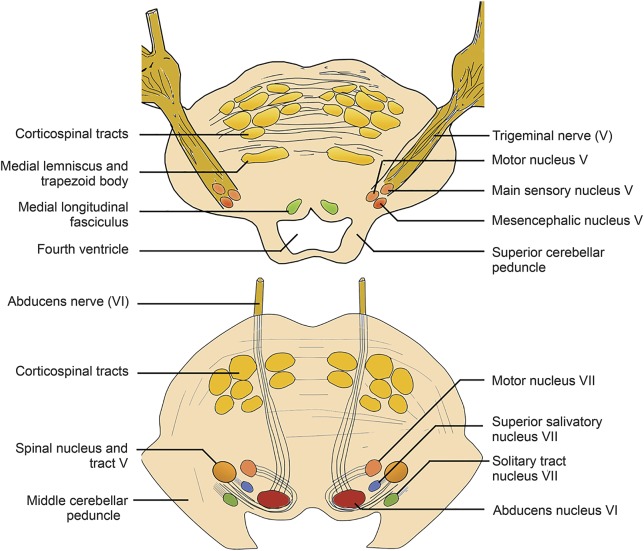
Anatomy of the trigeminal nerve and nuclei in the pons Two cross-sections are shown in the pons at the level of the trigeminal nerve, main sensory, and mesencephalic trigeminal nuclei (top) and spinal trigeminal nucleus and tract, superior salivatory nucleus, and solitary tract (bottom).

Our tractography results show that the DBS-activated area posterior to the hypothalamus in the ventral tegmentum lies on a tract that connects the hypothalamus, prefrontal, and mesial temporal regions anteriorly with brainstem areas in the proximity of the parabrachial nuclei, nucleus of the solitary tract, periaqueductal gray, and ending in the region of the trigeminal nucleus and tract and the superior salivatory nucleus (figures 2 and 3).

Although this finding does not explain the mechanism of action of DBS, it confirms the relevance of the target site by means of its connections to anatomically relevant brainstem areas. One possibility is the exertion of a top-down antinociceptive effect; another possibility is modulation of the trigeminal parasympathetic reflex, commonly activated in primary headache disorders^33^ and thought to mediate the cranial autonomic symptoms in CH.^34^ This pathway can be triggered by hypodermic capsaicin injection in the first trigeminal nerve division area^35^ and a variety of trigeminal nociceptive triggers.^26^ Nociceptive trigeminal activation, in the first division of the trigeminal nerve, is relayed into the spinal trigeminal nucleus and the C1/C2 dorsal horns (i.e., the trigeminocervical complex),^36^ which has a reflex connection to the superior salivatory nucleus in the pons.^37^ The output is then carried via the parasympathetic pathway of the facial nerve through the geniculate ganglion within the greater superficial petrosal nerve^38^ to the sphenopalatine ganglion.^26,39^

It must be noted, however, that the pain and the autonomic phenomenon can at times occur independently,^6^ especially in patients taking preventive medications, suggesting either anatomically separate pathways, albeit partly, or different activation thresholds mediating these 2 features.^39,40^

The largest reduction in HAL after VTA-DBS in patients with medically refractory CCH appears to correspond to activation in an area 6 mm lateral, 2 mm posterior, and 1 mm inferior to the MCP. Active contact DBS activation in responders lies on the THT, connecting the trigeminal system and other brainstem nuclei linked with nociception and pain modulation with the hypothalamus and prefrontal and mesial temporal areas. With only 1 nonresponder, it was not feasible to have a group comparison between responders and nonresponders to show whether alternative streamlines would be associated with a negative outcome.

## GLOSSARY

AC-PC: anterior commissure–posterior commissure
CCH: chronic cluster headache
CH: cluster headache
DBS: deep brain stimulation
HAL: headache load
MCP: midcommissural point
MNI: Montreal Neurological Institute
MPRAGE: magnetization-prepared rapid gradient-echo
THT: trigeminohypothalamic tract
VTA: ventral tegmental area
